# Cultural Dance Program Improves Hypertension Management for Native Hawaiians and Pacific Islanders: a Pilot Randomized Trial

**DOI:** 10.1007/s40615-015-0198-4

**Published:** 2015-12-22

**Authors:** Joseph Keawe‘aimoku Kaholokula, Mele Look, Tricia Mabellos, Guangxiang Zhang, Mapuana de Silva, Sheryl Yoshimura, Cappy Solatorio, Thomas Wills, Todd B. Seto, Ka‘imi A. Sinclair

**Affiliations:** 1 Department of Native Hawaiian Health, University of Hawaii, John A. Burns School of Medicine, 677 Ala Moana Blvd, Ste 1016B, Honolulu, HI 96813 USA; 2 Halau Mohala ‘Ilima, 1110 A‘alapapa Drive, Kailua, HI 96734 USA; 3 Kokua Kalihi Valley Comprehensive Family Center, 2239 N. School St., Honolulu, HI 96819 USA; 4 Kula No Na Po‘e Hawai‘i, 2150 Tantalus Drive, Honolulu, HI 96813 USA; 5 Cancer Prevention and Control Program, University of Hawaii Cancer Center, 701 Ilalo Street, Honolulu, HI 96813 USA; 6 The Queen’s Medical Center, 1301 Punchbowl Street, Honolulu, HI 96813 USA; 7 Department of Epidemiology, University of Washington, 1100 Olive Way, Ste 1200, Seattle, WA 98101 USA

**Keywords:** Native Hawaiian and Pacific Islanders, Hypertension, Hula, Community-based participatory research, Blood pressure

## Abstract

**Objective:**

Native Hawaiians and Pacific Islanders (NHPI) bear an unequal burden of hypertension and cardiovascular disease. Hula, the traditional dance of Hawaii, has shown to be a culturally meaningful form of moderate-vigorous physical activity for NHPI. A pilot study was done in Honolulu, Hawaii, to test a 12-week hula-based intervention, coupled with self-care education, on blood pressure management in NHPI with hypertension in 2013.

**Method:**

NHPI with a systolic blood pressure (SBP) ≥140 mmHg were randomized to the intervention (*n* = 27) or a wait-list control (*n* = 28). Blood pressure, physical functioning, and eight aspects of health-related quality of life (HRQL) were assessed.

**Results:**

The intervention resulted in a reduction in SBP compared to control (−18.3 vs. −7.6 mmHg, respectively, *p* ≤ 0.05) from baseline to 3-month post-intervention. Improvements in HRQL measures of bodily pain and social functioning were significantly associated with SBP improvements in both groups.

**Conclusion:**

Using hula as the physical activity component of a hypertension intervention can serve as a culturally congruent strategy to blood pressure management in NHPI with hypertension.

**Trial registration:**

clinicaltrials.gov Identifier: NCT01995812

## Introduction

Hypertension, or chronic high blood pressure, is a serious public health concern in the USA, affecting 25 % of the adult population [1]. It is a major risk factor for coronary heart disease (CHD) and the single most important risk factor for stroke, accounting for nearly 50 % of ischemic strokes [2]. Native Hawaiians and other Pacific Islanders (NHPI) bear an unequal burden of hypertension and its consequences. Compared to Whites, they are 70 % more likely to have hypertension, three and four times more likely to have CHD and stroke, respectively, and develop these diseases a decade sooner on average [3–5]. NHPI are also less likely to receive adequate hypertension treatment than Whites, leading to a greater risk of CHD and stroke [6, 7].

Western medical and behavioral approaches to hypertension management may not be as attractive to many NHPI than those that also include culturally relevant strategies, such as exercise programs based on their own cultural norms, and are sensitive to their cultural values and preferred modes of living [8–11]. Previous studies of NHPI found that they prefer traditional Pacific approaches to healing and treatment modalities that account for their unique spiritual and cultural values and delivered within a familiar community setting [11–14]. Many NHPI also have a distrust toward Western medicine that make them reluctant to participate in medical-focused health promotion programs [13]. Thus, hypertension interventions based on culturally meaningful health promotion strategies could lead to more NHPI participating in such interventions and lead to sustainable behavior change because of greater relevance to their lived cultural context. Given the higher risk of CHD and stroke in NHPI than other ethnic groups, and at younger ages, it is imperative that alternative and innovative strategies to hypertension management be developed and tested.

Physical activity is a major part of behavioral lifestyle interventions to both prevent and treat hypertension [15, 16]. Increased physical activity is associated with reduction in systolic blood pressure (SBP) of 5 to 10 mmHg and in diastolic blood pressure (DBP) of 1 to 6 mmHg in hypertensive patients [17], which is comparable to the effects of sodium restriction [18] and weight reduction [19]. A reduction of 5.5 mmHg in SBP and 3.0 mmHg in DPB has been found to lower the risk of CHD by 15 %, stroke by 27 %, and all-cause mortality by 7 % [20, 21]. The effects of physical activity can be magnified when coupled with other lifestyle changes, such as sodium reduction and weight loss, and are likely synergistic with pharmacologic interventions [22].

Despite the benefits of physical activity, many NHPI find it difficult to engage in regular physical activity because of socioeconomic barriers [23] and because many physical activity programs are individually based and less appealing, e.g., walking on a treadmill [24, 25]. Previous studies find that NHPI desire culturally relevant physical activity programs that are consistent with their ethno-cultural values, such as group-based activities that foster a mind-body connection [11, 12]. Tai chi, a mind-body physical activity originating out of Chinese martial arts, has been found to reduce SBP by 9.3 to 14.3 mmHg and DPB by 6.0 to 7.2 mmHg among hypertensive patients [26].

Hula is the traditional dance of Native Hawaiians, the indigenous people of Hawaii, and a hallmark of Hawaiian culture today. It is performed by men and women of all ages. Much like tai chi, hula appeals to a wide range of people from different ethnic groups and has spread beyond Hawaii to places such as Japan, Mexico, and Europe [27]. Originally performed to convey history, spiritual beliefs, and one’s connection to the natural world, hula is now practiced as a form of cultural and creative expression. The dances of hula are comprised of specific controlled rhythmic movements that illustrate the meaning or poetry of the accompanying songs or chants [27, 28]. They can vary in intensity and duration depending on the choreography of the dance, tempo of the music, and skill level of the dancer and can be modified to accommodate people who have physical limitations.

Hula has been found to be suitable as a form of physical activity with metabolic equivalents (METs) of 5.7 (range 3.17–9.77) and 7.55 (range 4.43–12.0) for moderate-intensity and high-intensity physical activity, respectively [29]. METs ranging from 3.0 to 6.0 (i.e., expends 3.5 to 7 kcal/min) are indicative of moderate physical activity while greater than 6.0 (i.e., expends >7 kcal/min) are indicative of vigorous physical activity [30]. As suggested of tai chi [31, 32], hula may also have stress-reducing benefits because it includes music and rhythmic movements and often performed in groups, which could provide social support [11]. Some studies have found that social support [33, 34] and music therapy [35] improves hypertension management. Thus, hula combines several aspects—moderate physical activity, social support opportunities, and other stress-reducing qualities—important to hypertension management.

To improve hypertension management in NHPI, the academic and community researchers of the Hula Empowering Lifestyle Adaptations (HELA) Project designed a hypertension program with hula as its physical activity component, called *Ola Hou i ka Hula* (Ola Hou) translated as “renewed life through hula” [8, 10]. We conducted a pilot randomized controlled trial (RCT) to test both feasibility and efficacy of Ola Hou in reducing the blood pressure in a community sample of NHPI with physician-diagnosed hypertension. We hypothesized that Ola Hou would lead to greater reductions in blood pressure when compared to a wait-list intervention control group. We further hypothesized that Ola Hou would improve both physical and social functioning and that these improvements would be associated with blood pressure reductions.

## Method

### Study Design

**Fig. 1:**
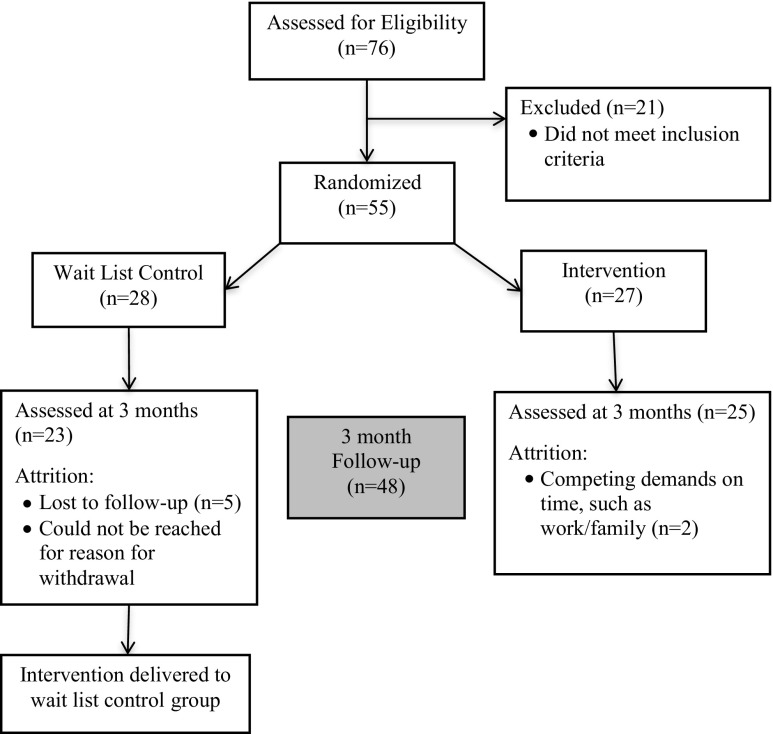
CONSORT diagram of Ola Hou i ka Hula study participation

We conducted a pilot study using a 2-arm RCT with a wait-list control group to test the effects of our 12-week Ola Hou intervention on blood pressure in NHPI with physician-diagnosed hypertension. This pilot study was conducted between October 2012 and June 2013. Figure 1 presents the CONSORT diagram and depicts the overall study design. We used a community-based participatory research (CBPR) approach in designing and implementing this study [8]. Our community investigators included a *kumu hula* (hula expert) and two NHPI community leaders, while our academic investigators included a cardiologist, psychologist, and public health researchers. Because this was a pilot study to also determine if the intervention was feasible for the community partners to implement in their respective community settings using their own community resources (e.g., kumu hula), we limited the data collection and measures to those essential to establish efficacy. Thus, we relied on randomization to balance participant unmeasured characteristics that might influence the outcomes, such as difference in hypertension medications prescribed and used.

### Participants

Participants with diagnosed hypertension were recruited from two community-based organizations: a community health center primarily serving immigrant Pacific Islanders and a Native Hawaiian residential community center. The enrollment goal was 60 NHPI with 1:1 randomization within each community site. Eligibility criteria were (1) under a physician’s care for ≥6 months for hypertension, (2) continued to have indications of hypertension (SBP >140 or >130 if have co-morbid diabetes), (3) ≥21 years of age, and (4) independently ambulatory. Exclusion criteria were (1) being prescribed more than four hypertension medications, (2) severe cognitive dysfunction precluding informed consent and understanding the intervention protocols, and/or (3) pregnancy at time or during the study period.

### Primary Outcome Measures

The primary outcome or endpoint of this study was blood pressure (mmHg), which was measured with an automatic blood pressure machine (Omron©HEM-907XL, Omron Healthcare, Palatine, Illinois). We used standardized protocols for obtaining blood pressure [36, 37]. For each assessment, SBP and DBP were measured in the sitting position and the participant was asked to sit quietly for 2–5 min before measuring blood pressure. Both SBP and DBP were measured three times from each participant at each assessment occasion, and the average of the three at each was used for data analyses. Only SBP was considered as the primary outcome for this study since participants’ eligibility was based only on their SBP being in the hypertensive range. However, DBP was also collected and analyzed.

### Secondary Outcome Measures

A secondary outcome or endpoint was physical functioning as measured by the 6-min walk test (6MWT), which measures the distance a person is able to walk in 6 min [38]. Participants were asked to perform the 6MWT using a fixed lap distance of either 60 or 100 ft. Each participant was instructed to walk as briskly as possible (without running) for 6 min. Instructions and prompts for the 6MWT are based on standardized protocols. It has been found to be a valid test to estimate physical functioning [38] and has been used in other RCT with NHPI [39].

Another secondary outcome or endpoint was health-related quality of life (HRQL) as measured by the Medical Outcomes Study 12-Item Short Form Health Survey (SF-12) [40]. The SF-12 has eight subscales that assess facets of a person’s HRQL: (1) physical functioning, (2) role physical functioning, (3) bodily pain, (4) general health, (5) vitality, (6) social functioning, (7) role-emotional functioning, and (8) mental health. Each subscale score, after transformation, ranges from 0 to 100, with a higher score indicating a better health status. The SF-12 has been extensively used and found to be a reliable and valid measure of HRQOL across different populations [41].

### Demographic and Body Mass Index

Demographic variables and body mass index (BMI) were assessed for baseline comparison to ensure balanced randomization and for possible statistical adjustments in case of an imbalance. Demographic variables were collected using a brief questionnaire asking for respondent’s age, birthdate, sex (male or female), ethnicity (Native Hawaiian, other Pacific Islander, or other non-Pacific Islander ethnic groups), past experience dancing hula (yes or no), history of heart disease (e.g., heart attack or heart failure), history of other medical conditions (e.g., diabetes), and if they were using any prescribed hypertension medication (yes or no). Weight in kilograms and height in centimeters were measured of each participant to calculate BMI (i.e., kg/m^2^).

### Procedures and Intervention Description

The University of Hawaii Committee on Human Studies approved the study protocol. Community health workers recruited potential participants through flyers posted in their respective community settings, community health fairs, and by word of mouth. After individuals were screened, enrolled, and completed their baseline assessment, they were randomized 1:1 within each of the two community-based organizations to either the Ola Hou intervention or to the wait-list intervention control group. Participants in both study arms were instructed to continue receiving usual care from their physician and that their participation in this study did not replace the need for routine medical care. All participants obtained their physicians’ approval for their participation in moderate physical activity.

Participants randomly assigned to the wait-list group did not receive the Ola Hou program and its educational materials, and no contact was made with them during the time they were on the wait-list. However, they were not restricted from seeking out other interventions, educational materials, or help on their own. They were free to perform, on their own, any type of exercise during this time and participate in any other health promotion program.

Participants assigned to the Ola Hou program received 3 h of hypertension education and 12 weeks of hula instruction and training consisting of two 60-min classes per week: The 3-h Ola Hou hypertention education curriculum was previously developed and culturally adapted for NHPI based on the Heart Failure Society of America’s educational guidelines and modules [42]. The curriculum included four modules: (1) signs and symptoms of hypertension, (2) managing medication, (3) heart healthy eating to include sodium reduction, and (4) physical activity and managing negative emotions. Community health workers led the education sessions and brief interactive activities, such as cooking demonstrations of healthy recipes of NHPI ethnic foods and practicing of self-monitoring and stress management strategies. This education curriculum was delivered in six, 30-min sessions immediately after the 60-min hula sessions from week 2 through week 7. Each hula class consisted of 12 to 15 participants, which provided them with the opportunity to develop and engage in a social support network. The specific dances used in this study were evaluated and found to be an appropriate level of physical activity based on national recommendations [43, 44] and for individuals with limited mobility and fitness [8]. Classes were delivered by a kumu hula and included the phases and goals described in Table 1.

**Table 1:** Design of Ola Hou physical activity program

Phase	Duration	Goals
Warm-up	5–15 min	• Promote normal range of motion • Stretches focusing on legs, arms, lower back • Low-level, aerobic activity at 25–40 % MPHR
Conditioning	20–40 min	Intensity • Range 40–85 % VO_max_ or 50–70 % MPHR • Training intensity = (40 + [2 × Max METs]) % • RPE: between 12 and 16 on Borg scale^a^ • Target HR = (HR reserve × training intensity [%]) + HR_resting_
Cool-down	3–10 min	• Low-level, aerobic activity to allow BP and HR to return to resting level

*MPHR* maximum predicted heart rate, *VO*_*max*_ maximal oxygen consumption, *METs* metabolic equivalents, *RPE* rating of perceived exertion, *HR* heart rate, *BP* blood pressure

^a^The Borg scale is commonly used in cardiac rehabilitation as a subjective measure of physical exertion. The participant gives a subjective score between 6 and 20 during exercise to indicate his or her level of physical intensity [45]

The design of the Ola Hou program was informed by the social cognitive theory [45], which has been used successfully to inform many types of physical activity interventions [46]. It is primarily based on the principle of reciprocal determinism, referring to the bidirectional behavior-environment interaction on influencing behavior. It posits that a person’s knowledge or behavior acquisition is directly related to the observation of others (or models) within his or her context of social interactions and experiences. It is through these social observations and interactions that a person’s self-efficacy to make and sustain behavior change is achieved and reinforced.

All participants, both those in the intervention and wait-list control, were assessed by study staff using standardized protocols to obtain clinical and demographic and psychosocial information through in-person interviews. Assessments were performed at baseline and 3-months post-intervention. All participants received a $25 gift card after the completion of each assessment period. Intervention participants received incentives such as cooking supplies, bags, and water bottles at various time points during the Ola Hou program.

### Statistical Analysis

The baseline demographics, SBP, DBP, 6MWT, and SF-12 subscale scores were summarized by descriptive statistics: means (M) and standard deviations (SD) for continuous variables (i.e., age, BMI, SBP, DBP, 6MWT, and SF-12 subscale scores) and frequencies and proportions for categorical variables [i.e., sex (female vs. male), ethnic group (Native Hawaiian, other Pacific Islander, or other ethnic group), heart disease (no vs. yes), other medical conditions (no vs. yes), and prescribed hypertension medication (no vs. yes)]. Comparisons between the two study arms at baseline on the aforementioned variables were conducted using two sample *t* test, chi-squared, or Fisher’s exact tests, as appropriate. The retention rates between two groups were evaluated by Fisher’s exact test.

Multivariable linear regression modeling were used to examine change in our primary endpoints of SBP and DBP (primary outcomes), separately, from baseline to 3-month assessment (i.e., 3-month BP − baseline BP; continuous change variable) between study arms while adjusting for age, baseline blood pressure, and baseline characteristics (i.e., presence of heart disease or not; see Table 2) that were notably (*p* < 0.10) different between groups. Age was adjusted, as recommended for hypertension outcome studies [1, 15]. It is also convention when examining the effects of a RCT intervention to adjust for the baseline value of the primary outcome when change from baseline is the primary endpoint. Both intention-to-treat and complete case analyses were done for SBP and DPB each using multivariable linear regression modeling.

**Table 2:** Baseline characteristics of participants by study group

Variable	Ola Hou (*n* = 27)	Control (*n* = 28)	Group differences (*p* value)^a^
Age in years, *M* (*SD*)	55 (10)	55 (12)	0.90
Female, *n* (%)	25 (93)	22 (79)	0.26
Ethnic group, *n* (%)			1.00
Native Hawaiian	13 (48)	14 (50)	
Other Pacific Islander	13 (48)	12 (43)	
Other ethnic group	1 (4)	2 (7)	
Danced hula, *n* (%)	11 (41)	7 (25)	0.26
Heart disease, *n* (%)			
Heart attack	0 (0)	2 (7)	0.50
Heart failure	0 (0)	1 (4)	1.00
High cholesterol	12 (44)	19 (68)	0.11
No heart disease	15 (56)	8 (29)	0.06
Other medical conditions, *n* (%)			
Diabetes	16 (59)	15 (54)	0.79
Other medical conditions	7 (26)	7 (25)	1.00
No other medical conditions	6 (22)	7 (25)	1.00
Prescribed hypertension medications, *n* (%)	20 (74)	25 (89)	0.18
BMI, kg/m^2^, *M* (SD)	36 (7)	39 (6)	0.11
SBP, mmHg, *M* (SD)	148 (14)	143 (12)	0.16
DBP, mmHg, *M* (SD)	85 (12)	83 (13)	0.71
6MWT, ft, *M* (SD)	1264 (191)	1336 (261)	0.26
SF-12 subscales, *M* (SD)^b^			
Physical functioning	46 (40)	50 (42)	0.74
Role physical functioning	48 (28)	41 (33)	0.43
Bodily pain	63 (28)	71 (17)	0.23
General health	48 (20)	56 (20)	0.15
Vitality	57 (25)	63 (28)	0.48
Social functioning	52 (32)	66 (31)	0.10
Role-emotional functioning	51 (34)	56 (38)	0.62
Mental health	58 (20)	63 (17)	0.28

*M* mean, *SD* standard deviation, *BMI* body mass index, *SBP* systolic blood pressure, *DBP* diastolic blood pressure

^a^Group difference *p* values based on two sample independent *t* tests and chi-squared or Fisher’s exact tests, as appropriate

^b^SF-12 subscale scores are calculated as a percentage (0 to 100 %) with higher scores indicating better health and well-being

Intention-to-treat analyses were performed for all participants randomized, with missing values at 3-month assessments imputed by baseline observation values carried forward. Complete case analyses were also conducted, which included only the participants with both baseline and 3-month assessment data available. Thus, seven participants with only baseline assessment data but without 3-month data were excluded in the complete case analyses.

To further evaluate the clinical impact of the Ola Hou program on hypertension control, participants in both study arms were categorized based on those with a reduction of SBP ≥ 10 mmHg versus those without. A reduction of ≥10 mmHg in SBP over 3 months was regarded as being clinically significant (i.e., reducing the risk for CHD and stroke) based on the findings of previous studies [20, 21]. Chi-squared (χ^2^) analysis was used to compare between participants of the two study arms based on this SBP categorization.

Multivariable linear models were also used to examine the change in our secondary endpoints, separately, from baseline to 3-month assessment (i.e., 3-month value − baseline value) between study arms while adjusting for age, baseline value of the relevant secondary endpoint, and baseline characteristics (i.e., presence of heart disease or not; see Table 2) that were notably (*p* < 0.10) different between groups. Both intention-to-treat and complete case analyses were also done for these analyses.

We also examined the association between the changes in secondary endpoints and the changes in SBP (the primary endpoint) from baseline to 3-month assessment in the entire sample combined. These associations were evaluated by Pearson correlation coefficients. These analyses were done to determine what physical functioning and HRQL variables significantly co-varied with SBP regardless of study arm.

Formal sample size calculations were not performed for this pilot study. However, sample size was estimated based on a review of literature of similar mind and body physical activities used in hypertension interventions [31, 32, 47, 48]. Study data were collected and managed using REDCap electronic data capture tools [49] hosted at the University of Hawaii John A. Burns School of Medicine. All statistical analyses were performed using SAS software version 9.3 (SAS Institute Inc., Cary, NC). A two-sided *p* value ≤0.05 was considered statistically significant.

## Results

### Participants

Table 2 summarizes the baseline characteristics, which were comparable for the most part between the participants of the two study arms. For both study arms, the mean age was 55 years (SD = 10 for Ola Hou and 12 for wait-list control participants) and a majority were NHPI (95 %) and female (85 %). More participants in the Ola Hou program (41 %) compared to control (25 %) had previously danced hula and had no previous history of heart disease (56 and 29 %, respectively), but these differences were not statistically significant. However, the difference between arms on the number of participants with “no heart disease” did approach significance (*p* = 0.06). Both study arms were balanced in regards to having “other medical conditions,” to being on “prescribed hypertension medications,” and to BMI, SBP, DBP, 6MWT, and scores on the SF-12 subscales.

### Retention

Of the 55 participants assigned to the two study arms, 48 (87 %) completed the 3-month post-intervention assessment. There was no significant difference (*p* = 0.42) in attrition between the intervention arm (*n* = 2, 7 %) and the wait-list control arm (*n* = 5, 18 %) at 3-month assessment.

### Primary Outcome Analyses

Table 3 summarizes the results of the blood pressure outcomes by study arms. Ola Hou participants, compared to wait-list control participants, had greater reductions in their SBP in both the intention-to-treat analysis (−18.3 vs. −7.6 mmHg, respectively) and the complete case analysis (−19.8 vs. −9.2 mmHg, respectively) from baseline to 3-month assessment. After adjusting for age, heart disease status, and baseline blood pressure, there were significant differences at 3 months between the Ola Hou and the wait-list control group for SBP, in both the intention-to-treat [*F*(1, 50) = 4.19, *p* = 0.046] and complete case analyses for SBP [*F*(1, 43) = 4.24, *p* = 0.045]. There were no significant differences in DBP between the intervention and control participants from baseline to 3-month assessment. The invention group also had significantly more participants with at least 10 mmHg reduction in SBP at 3-month assessment than those in the wait-list control group [72 vs. 39 %, respectively; *χ* ^2^ (1, *n* = 48) = 5.26, *p* = 0.022].

**Table 3:** Intervention effects on blood pressure from baseline to 3-month follow-up^a^

	Baseline *M* (*SD*)	3 month *M* (*SD*)	Change ± *SE*	*F* test	Group differences (*p* value)^b^
Systolic blood pressure					
Intention-to-treat^c^				*F*(1, 50) = 4.19	0.046
Ola Hou (*n* = 27)	148.4 (14.2)	130.1 (17.3)	−18.3 ± 3.2		
Control (*n* = 28)	143.4 (11.8)	135.8 (18.0)	−7.6 ± 3.0		
Complete cases^d^				*F*(1, 43) = 4.24	0.045
Ola Hou (*n* = 25)	147.9 (13.4)	128.1 (15.1)	−19.8 ± 3.3		
Control (*n* = 23)	144.1 (10.4)	134.9 (18.2)	−9.2 ± 3.6		
Diastolic blood pressure					
Intention-to-treat^c^				*F*(1, 50) = 0.80	0.38
Ola Hou (*n* = 27)	84.6 (12.1)	79.0 (10.9)	−5.6 ± 1.6		
Control (*n* = 28)	83.4 (12.6)	80.5 (13.1)	−2.9 ± 2.0		
Complete cases^d^				*F*(1, 43) = 0.98	0.33
Ola Hou (*n* = 25)	84.2 (12.1)	78.1 (10.4)	−6.1 ± 1.7		
Control (*n* = 23)	83.7 (11.2)	80.2 (11.9)	−3.5 ± 2.4		

*M* mean, *SD* standard deviation, *SE* standard error

^a^Unadjusted descriptive means (SD) and changes (SE) are reported in the table

^b^Based on multivariable linear model with the blood pressure change (3-month value minus baseline value) as the dependent variable, adjusting for the baseline blood pressure value, age, and heart disease status (presence vs. absence)

^c^Intention-to-treat analyses with missing value of blood pressure at 3-month assessment imputed by individual participant’s baseline observation value carried forward

^d^Complete case analyses included only participants with both baseline and 3-month assessment data available (*n* = 48)

### Secondary Outcome Analyses

Table 4 summarizes the results of the physical functioning and self-reported HRQL outcomes by study arms. Adjusting for age, heart disease status, and baseline value of the secondary outcome variable, there were no significant differences between the study arms on the 6MWT and SF-12 subscales in change from baseline to 3-month assessment based on either intention-to-treat analysis or complete case analysis. However, it is important to note that greater average improvements on these variables were found in the Ola Hou participants compared to the wait-list control participants, and the difference between them on the bodily pain and social functioning scores did approach significance (*p* < 0.08). It is also important to note that on five of the eight, the scores on the SF-12 subscales (i.e., bodily pain, general health, vitality, social functioning, and mental health) worsened, on average, from baseline to 3-month assessment for the wait-list control group.

**Table 4:** Intervention effects on secondary outcomes at baseline and 3-month follow-up^a^

	Baseline, *M (SD)*	3 month, *M (SD)*	Change ± *SE*	*F* test	Group differences (*p* value)^b^
6-Min walk test, ft					
Intention-to-treat^c^				*F*(1, 50) = 0.003	0.96
Ola Hou (*n* = 27)	1264.4 (190.6)	1308.9 (278.7)	44.4 ± 37.3		
Control (*n* = 28)	1335.8 (261.0)	1351.1 (260.1)	15.3 ± 38.1		
Complete cases^d^				*F*(1, 43) = 0.04	0.84
Ola Hou (*n* = 25)	1263.6 (198.3)	1311.6 (289.8)	48.0 ± 40.3		
Control (*n* = 23)	1384.9 (252.1)	1403.5 (247.0)	18.6 ± 46.5		
Physical functioning					
Intention-to-treat^c^				*F*(1, 50) = 0.12	0.73
Ola Hou (*n* = 27)	46.3 (39.7)	53.7 (38.4)	7.4 ± 5.9		
Control (*n* = 28)	50.0 (41.9)	50.0 (36.0)	0.0 ± 6.2		
Complete cases^d^				*F*(1, 43) = 0.04	0.84
Ola Hou (*n* = 25)	50.0 (38.9)	58.0 (36.6)	8.0 ± 6.4		
Control (*n* = 23)	56.5 (42.1)	56.5 (34.7)	0.0 ± 7.5		
Role physical					
Intention-to-treat^c^				*F*(1, 50) = 0.38	0.54
Ola Hou (*n* = 27)	47.7 (28.0)	60.3 (24.9)	12.5 ± 5.1		
Control (*n* = 28)	41.1 (33.3)	52.2 (29.9)	11.2 ± 4.0		
Complete cases^d^				*F*(1, 43) = 0.10	0.75
Ola Hou (*n* = 25)	50.5 (26.9)	64.0 (26.3)	13.5 ± 5.4		
Control (*n* = 23)	46.7 (32.9)	60.3 (24.9)	13.6 ± 4.7		
Bodily pain					
Intention-to-treat^c^				*F*(1, 50) = 3.19	0.08
Ola Hou (*n* = 27)	63.0 (28.1)	72.2 (19.5)	9.3 ± 4.7		
Control (*n* = 28)	70.5 (16.7)	66.1 (22.8)	−4.5 ± 4.1		
Complete cases^d^				*F*(1, 43) = 3.62	0.064
Ola Hou (*n* = 25)	62.0 (29.0)	72.0 (19.5)	10.0 ± 5.0		
Control (*n* = 23)	70.7 (17.9)	65.2 (24.7)	−5.4 ± 5.0		
General health					
Intention-to-treat^c^				*F*(1, 50) = 0.75	0.39
Ola Hou (*n* = 27)	47.8 (20.1)	54.6 (18.4)	6.9 ± 3.6		
Control (*n* = 28)	55.7 (20.4)	53.8 (17.2)	−2.0 ± 3.2		
Complete cases^d^				*F*(1, 43) = 0.74	0.40
Ola Hou (*n* = 25)	46.8 (20.6)	54.2 (19.1)	7.4 ± 3.8		
Control (*n* = 23)	54.8 (22.4)	52.4 (18.8)	−2.4 ± 3.9		
Vitality					
Intention-to-treat^c^				*F*(1, 50) = 1.29	0.26
Ola Hou (*n* = 27)	57.4 (24.8)	60.2 (18.7)	2.8 ± 5.4		
Control (*n* = 28)	62.5 (27.6)	56.3 (23.2)	−6.3 ± 4.7		
Complete cases^d^				*F*(1, 43) = 1.65	0.21
Ola Hou (*n* = 25)	55.0 (23.9)	58.0 (17.3)	3.0 ± 5.8		
Control (*n* = 23)	59.8 (27.9)	52.2 (21.2)	−7.6 ± 5.8		
Social functioning					
Intention-to-treat^c^				*F*(1, 50) = 3.29	0.08
Ola Hou (*n* = 27)	51.9 (31.7)	63.0 (28.9)	11.1 ± 3.9		
Control (*n* = 28)	66.1 (31.3)	60.7 (30.8)	−5.4 ± 5.2		
Complete cases^d^				*F*(1, 43) = 1.75	0.19
Ola Hou (*n* = 25)	55.0 (30.6)	67.0 (25.7)	12.0 ± 4.1		
Control (*n* = 23)	75.0 (26.1)	68.5 (27.4)	−6.5 ± 6.3		
Role emotional					
Intention-to-treat^c^				*F*(1, 50) = 0.08	0.80
Ola Hou (*n* = 27)	51.4 (34.0)	58.3 (29.0)	6.9 ± 6.0		
Control (*n* = 28)	56.3 (38.0)	58.0 (32.8)	1.8 ± 5.0		
Complete cases^d^				*F*(1, 43) = 0.06	0.80
Ola Hou (*n* = 25)	54.5 (33.2)	62.0 (26.6)	7.5 ± 6.5		
Control (*n* = 23)	65.2 (34.7)	67.4 (26.8)	2.2 ± 6.1		
Mental health					
Intention-to-treat^c^				*F*(1, 50) = 1.06	0.31
Ola Hou (*n* = 27)	57.8 (20.0)	63.4 (17.7)	5.6 ± 3.6		
Control (*n* = 28)	63.4 (17.0)	62.5 (16.7)	−0.9 ± 2.2		
Complete cases^d^				*F*(1, 43) = 0.91	0.35
Ola Hou (*n* = 25)	58.5 (20.6)	64.5 (17.9)	6.0 ± 3.9		
Control (*n* = 23)	65.8 (17.8)	64.7 (17.5)	−1.1 ± 2.7		

*M* mean, *SD* standard deviation, *SE* standard error

^a^Unadjusted descriptive means (SD) and changes (SE) are reported in the table

^b^Based on multivariable linear model with the relevant variable change (3-month value minus baseline value) as the dependent variable, adjusting for the variable’s baseline value, age, and heart disease status (presence vs. absence)

^c^Intention-to-treat analyses with missing value at 3-month assessment imputed by individual participant’s baseline observation value carried forward

^d^Complete case analyses included only participants with both baseline and 3-month assessment data available (*n* = 48)

### Association between SBP and Secondary Outcomes

For both Ola Hou and wait-list control participants, improvements in bodily pain [*r*(46) = −0.32, *p* = 0.03] and social functioning scores [ *r*(46) = −0.50, *p* = 0.0003] were significantly associated with improved change in SBP from baseline to 3-month assessment (data not shown in tables). None of the other SF-12 subscale scores were significantly associated with the improved change in SBP from baseline to 3-month assessment.

## Discussion

We examined the efficacy of a hula-based intervention in improving blood pressure control, physical functioning, and HRQL in a community-based sample of NHPI with a confirmed diagnosis of hypertension. In partial support of our hypothesis, we found that the hula-based Ola Hou program led to significantly greater reductions in SBP and notable improvements in bodily pain when compared to those randomized to the wait-list intervention control group. Improvements in both bodily pain and social functioning were significantly associated with improvements in SBP in both groups. However, there were no statistically significant differences between the intervention and control group on measures of physical functioning and HRQL.

We found that the Ola Hou Program led to a greater reduction in SBP (−9.8 mmHg) over the 3-month intervention period. This improvement is comparable to other studies of physical activity-based interventions [17] and the use of tai chi [26] for hypertension management. The hula-based intervention led to a reduction in SBP of 10 mmHg or greater for 72 % of the intervention participants compared to 39 % of those in wait-list control. A 5 mmHg reduction in SBP has been found to lower a person’s risk of ischemic heart disease by 21 %, stroke by 34 %, and all-cause mortality by 7 % [20, 50]. This magnitude of reduction in SBP exceeds the reduction typically seen with antihypertensive medication monotherapy [51]. We did not find significant improvements in DBP in this study, which is likely due to the fact that we based eligibility for participation on systolic hypertension (>140 mmHg); the participants, on average, had a DBP under 90 mmHg at baseline.

The association between improved social functioning and decreased SBP for NHPI suggests another mechanism by which blood pressure can be affected, beyond the effects of physical activity itself. We found that social functioning was associated with SBP improvements for both groups. When comparing between groups, we found that social functioning, on average, improved for the intervention participants but slightly worsened over time for the control participants, although this difference was short of being statistically significant. However, it does provide support for the reciprocal determinism principle of the social cognitive theory [45]; that is, the social context can serve to influence a person’s physical activity and other behavior changes [46]. We hypothesized that social functioning would improve as a result of participating in a hula-based intervention because hula is typically taught and practiced in a group setting and emphasizes synchronization in dancing. In addition, the hula training promotes the Hawaiian value of interconnectedness between individuals and fosters a family-like environment. It also emphasizes a mind-body approach by incorporating an understanding of the historical and cultural meaning behind the songs or chants that accompany the dance. The gestures and movements of hula are directly related to the words and their meaning, often metaphorically, which encourages coordination of mind and body to achieve a state of “being in the moment” and attuned to the symbolic meaning of one’s movements.

As previously discussed, hula was found to achieve the METs expected of a moderate (5.7) to vigorous intensity (7.6) exercise and thus hypothesized to have benefits for hypertension management for NHPI [29]. The finding of our study supports this notion that hula, coupled with hypertension self-care education, can be effectively used for hypertension management among NHPI. Hula as a physical activity may also have synergistic effects with social support and social functioning and with the use of music and rhythmic motions that enhance hypertension control. Previous studies have found that social support [33, 34] and music [25] can lower blood pressure and may operate by stress reduction to lower sympathetic arousal [52, 53]. Future studies are needed to elucidate the mechanism by which hula, as a mind-body physical activity approach, works to improve blood pressure in hypertension patients.

Notwithstanding the positive outcomes of our study, there are several methodological limitations worth noting. Aside from the 6MWT, we used subjective self-report measures of physical, emotional, and social functioning that are susceptible to response bias. However, it is unlikely that response bias was a problem in this study given that the more objective 6MWT along with most of the physical functioning self-report measures did not show significant improvements over the course of the intervention, although there was a trend toward improvement in most measures. We included NHPI with hypertension diagnosis based only on SBP. The effects of a hula-based intervention on DBP have yet to be determined. Another important limitation of our study is the absence of hypertension medication information to control for in the analysis. It is possible that those who participated in the Ola Hou program became more aware of their need to comply with their prescribed medication regimen. Although improved medication compliance is a desired outcome of the intervention, we were unable to determine if medication type or use may have influenced the differences we observed between study groups because of the pilot nature of our study. However, randomization should have balanced the two groups regarding medication use.

Overall, our study provides the strong preliminary evidence needed to more definitively test the effects of hula as a cultural dance to improve not only physical activity but other factors important to hypertension management in Native Hawaiians and Pacific Islanders. It also provides support for the investigation of other cultural forms of physical activity as the means to increase participation in health promotion interventions and the uptake and maintenance of regular physical activity that is culturally meaningful and sustainable in real-world settings. Finally, our study suggest multiple pathways to improving hypertension management in high-risk minority populations that is worthy of further investigation, such as comparing the relative effects of social functioning versus physical activity, in improving blood pressure control.
